# Fractional radiofrequency‐assisted delivery of fluorescent tracer dyes in ex vivo and in vivo skin: A proof‐of‐concept investigation using confocal microscopy

**DOI:** 10.1111/srt.13253

**Published:** 2023-02-07

**Authors:** Gabriela Lladó Grove, Rikke Louise Christensen, Merete Haedersdal, Vinzent Kevin Ortner

**Affiliations:** ^1^ Department of Dermatology Copenhagen University Hospital Copenhagen Denmark

**Keywords:** confocal microscopy, drug delivery, energy‐based device, ex vivo porcine skin, fluorescence microscopy, fluorescence trace dye, fractional radiofrequency, in vivo human skin, skin remodeling, tissue interaction

Energy‐based device‐assisted drug delivery is a growing trend in dermatology, building on the concept of laser‐assisted drug delivery to achieve enhanced passive diffusion of topically applied compounds through laser channels.^1^, ^2^ Much like an ablative fractional laser, fractional radiofrequency (FRF) creates grids of microthermal zones (MTZ) consisting of ablative craters surrounded by coagulation zones.^2^, ^3^ FRF‐devices have been successfully used in the treatment of various skin conditions including scars, rhytids, and hyperpigmentation.^4^, ^5^, ^6^

In this descriptive proof‐of‐concept investigation, we sought to visualize FRF‐induced tissue interactions and provide the first pre‐clinical findings on FRF‐assisted delivery of fluorescent tracer dyes in skin.

The experimental study set‐up included ex vivo porcine skin and healthy in vivo human skin. A commercially available FRF‐device, Venus VivaMD (Venus Concept Inc, Toronto, Canada), was used. For both the ex vivo and the in vivo models, the device's maximal combined ablation and coagulation (271 V, 30 ms) settings were used with its standard high‐energy applicator tip (80‐pin tip; 124 mJ/pin).

For ex vivo interventions, nine skin sites were examined. Immediately after FRF‐treatment, the treated skin (*n* = 6) and the untreated control skin (*n* = 3) samples were placed in a Franz‐cell setup (PermeGear Inc.). The setup consisted of a receiver chamber with 5.5 ml phosphate buffered saline (PBS), a donor chamber with 0.5 ml solution containing ATTO‐647N, a fluorescent dye (carboxylic acid, λex = 646 nm, λem = 664 nm; ATTO‐TEC), and distilled water (0.05% dimethyl sulfoxide) to a 10 μM concentration. After 20 hours incubation, 8 mm punch biopsies for 100 μm vertical cryosections were mounted on a bimodal ex vivo confocal microscope (EVCM; Vivascope2500, MAVIG GmbH) for combined reflectance (RCM, λem = 638 nm) and fluorescence microscopy (FCM, λem = 638 nm for ATTO‐647N). For improved visualization of FRF‐tissue interactions, acridine orange (AO) was applied for digital HE‐staining (RCM: 638 nm, AO‐FCM: 488 nm).^7^

In the experimental investigation of in vivo human skin, after informed consent, 30 skin sites were examined, including incubated FRF‐treated (*n* = 19) and untreated control (*n* = 7) samples, as well as nonincubated FRF‐treated autofluorescence controls (*n* = 4). Incubation was performed with an aqueous sodium fluorescein (NaFl, Combiflure, MW = 376.27 Da, 300 μl) solution for 2.5 hours in a customized skin‐adhesive well of occlusive dressing (Tegaderm [3M], Duoderm [ConvaTec]). We employed an in vivo RCM‐FCM (488‐nm fluorescence, 785‐nm reflectance) device (Vivascope Multilaser 1500, MAVIG GmbH) and collected stacks of horizontal images (VivaStack).

Morphologic changes and local skin reactions (LSRs) were described qualitatively. Fiji Image J was used for qualitative description of FRF‐tissue interactions and quantitation of fluorescence signal intensity (FI) in images of in vivo skin. Average FI per field view (500 × 500 μm) in each test area was measured per stack, consisting of 20 slices of 3 μm each, corresponding to a total depth of 60 μm. Statistics were performed in SPSS Statistics version 25 (IBM Corp.) for describing the FI (arbitrary units, AU).

In the ex vivo porcine skin, subclinical FRF‐tissue interactions were visualized by EVCM. Digitally‐stained cross‐sectional EVCM‐images showed surface indentations and a slight increase in hyperreflectivity corresponding to a stronger digital Eosin signal. Focally enhanced uptake of ATTO‐647N in FRF‐treated sites was visible as pockets of increased FI in the epidermis and upper dermis (Figure 1). In control images, ATTO‐647N signal appeared as a homogeneous layer, strictly confined to stratum corneum and epidermis.

**FIGURE 1:**
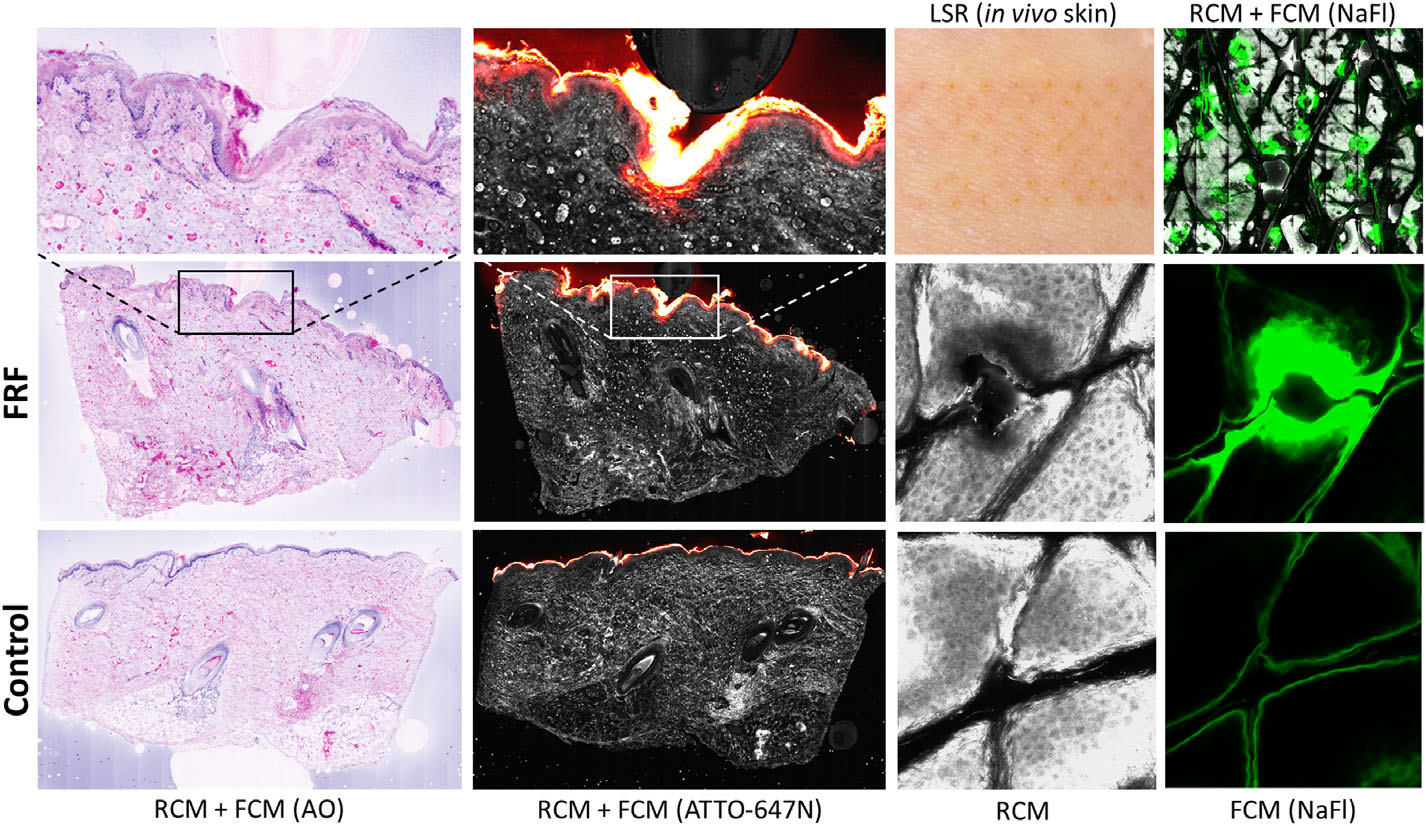
Confocal microscopy images of ex vivo porcine and in vivo human skin exposed to fractional radiofrequency (FRF) pretreatment and fluorescent tracer dyes. In ex vivo porcine skin (columns 1 and 2 from the left) combined RCM + FCM demonstrates superficial damage in digitally‐stained AO‐labeled images and a focal increase of ATTO‐647N in the MTZ. In in vivo human skin (columns 3 and 4 from the left), the LSRs and MTZ‐grid pattern are visualized clinically and with RCM + FCM, and the horizontal diffusion of NaFl is shown in the FRF‐treated skin sites at a tissue depth of 24 μm. AO, acridine orange; FCM, fluorescence confocal microscopy; LSR, local skin reaction; MTZ, microthermal zone; NaFl, sodium fluorescein; RCM, reflectance confocal microscopy

In in vivo human skin, we observed an immediate clinical response in all FRF‐treated sites, and LSRs within the treatment areas were visible indentations as an MTZ‐grid pattern and mild transient erythema and edema (Figure 1). In RCM images, FRF‐tissue interactions appeared as cuboidal‐to‐cylindrical, hyporeflective surface indentations with sharply demarcated hyperreflective rim, similar to ablative laser‐induced MTZ with a central ablation zone surrounded by a coagulation zone.^7^ Increased peak signal intensity in the coagulation zone surrounding the signal‐free ablation zone was visualized together with a characteristic distribution of NaFI, progressively decreasing with depth. The mean NaFl signal intensity across all image stacks of FRF‐treated skin was 30.5 AU, which was significantly higher (*p* < 0.01) than the mean FI of 8.4 AU in the intact skin, equivalent to an FI‐increase of 263% with FRF‐treatment. No significant autofluorescence in the treated, nonincubated sites was detected.

This is the first proof‐of‐concept report of experimental FRF‐assisted delivery of fluorescent tracer dyes into skin, using combined RCM‐FCM. Enhanced uptake of two different fluorescence tracer dyes was visualized in all treated sites in both the ex vivo porcine skin and in vivo human skin models. These findings unfold the possibility to expand energy‐based device‐assisted drug delivery to also include FRF in a clinical setting. Additional studies assessing the vehicle compatibility, optimal settings, and clinical safety and efficacy are warranted before clinical implementation of FRF‐assisted drug delivery. Furthermore, comparative studies of FRF and other energy‐based devices for assisted drug delivery should be conducted in order to establish the optimal implementation of FRF in the field. In conclusion, our imaging investigation showed the impact of FRF‐induced skin barrier disruption on the locally enhanced penetration of topically applied compounds.

## CONFLICT OF INTEREST

MH has received equipment and research grant from Venus Concept Inc. Other authors have no conflict of interest to declare.

## ETHICS STATEMENT

Informed consent was obtained.

## Data Availability

The data that support the findings of this study are available from the corresponding author upon reasonable request.
